# Influence Exerted by Anæsthetics on the Brain and Nervous System

**Published:** 1868

**Authors:** 


### INFLUENCE EXERTED BY ANÆSTHETICS ON THE BRAIN AND NERVOUS SYSTEM.

This was the subject of a recent lecture by Dr. B. W. Richardson. The obvious fact that the motion of the heart and the movements of respiration continue in action while the rest of the body is under the narcotic effect during anæsthesia proves that the whole nervous system is not involved, and that the involuntary and semi-voluntary muscular mechanism is also not involved except when extreme and fatal symptoms are developed. What parts, then, are influenced by an anæsthetic? The idea was almost intuitive that the brain is the organ affected, and that the centers of consciousness are those chiefly held in abeyance. But to prove this as true, experiment was necessary. In proof, the lecturer took a large pigeon, narcotized it deeply with chloroform, and in this state passed through its body, from the head to the foot, a rapid intermittent induction current. The bird instantly rose from the table, extended its wings, opened its eyes, and seemed as if restored; the current was then stopped, and the bird was shown to be as deeply asleep and as powerless as before. Another bird was put to sleep by freezing the brain; and when utterly insensible, was subjected to the electrical shock in the same way, when it flew from the table into the room, where, breaking its connection with the battery, it dropped on the floor comatose, motionless, and as anæsthetized as before, in which condition it remained for many minutes. The lecturer, in these experiments, demonstrated that the anæsthetic action was localized in the cerebrum. His battery was like an outer brain, which supplied power without intelligence, and which, by the effects of its current, showed that all the muscular elements were ready for work, and only awaited the order from the brain. The lecturer next discussed the question, What, during the process of anæsthesia, leads to this change in the brain? Is there a chemical action on albumen? Is there pressure on brain matter? Is there deficient oxidation of the blood? Is there contraction of blood vessels, and diminished supply of blood from that cause? All these hypotheses were experimentally tested and negatived. It was admitted that, during extreme anæsthesia, there is reduced oxidation and a singular reduction of temperature. These changes are inevitable, because the anæsthetic vapours replace oxygen during their diffusion into blood; but the diminished oxidation is not the cause of the insensibility. In proof of this, Dr. Richardson showed an animal breathing an air in which the oxygen was reduced by addition of vapour of bichloride of methylene only to about 20 parts to the 100—viz., 4 cubic inches in 500. The result was that the animal, in the extremely reduced atmosphere, was quite unaffected, whilst the animal in the slightly reduced atmosphere was in the deepest narcotism. Then a correcting experimental test was adopted, and the bichloride was administered in an atmosphere containing an excess of oxygen, the oxygen being present in double its ordinary or natural proportion; the excess of oxygen exerted no perceptible obstacle to the anæsthesia. To determine whether there was contraction of blood vessels under anæsthetics, the lecturer had had recourse to transparent small trout; through their bodies, with the microscope and the one-inch lens, the blood vessels could be seen, and the corpuscles flowing through them. These animals can be narcotized readily by making them breathe water saturated with chloride of methylene or ether. In the narcotized condition, the vessels do not contract, but under the influence of ether, in the latter stages before death occurs, dilatation and regurgitation are observed. The latter is noticed, also, when chloride of methylene is used. With both reagents breathing, and vessel circulation cease before the heart's action. The lecturer concluded that anæsthetic vapours act directly upon nerve matter either by preventing the development of force or by stopping conduction. The latter hypothesis is supported by the fact, proved by experiment, that these vapours obstruct the conduction of heat and electricity.—*Medical Times and Gazette.*

